# The anti-allergic effect of *Lactobacillus plantarum* GUANKE on allergic rhinitis induced by either ragweed pollen or house dust mites in mice

**DOI:** 10.3389/fnut.2025.1678249

**Published:** 2025-11-10

**Authors:** Pei Zhao, Bin Zhang, Lian S. Zhao, Zhongli Yang, Ming D. Li

**Affiliations:** 1 College of Life Sciences, Shanxi Agricultural University-Taigu Campus, Jinzhong, China; 2 State Key Laboratory for Diagnosis and Treatment of Infectious Diseases, National Clinical Research Center for Infectious Diseases, Collaborative Innovation Center for Diagnosis and Treatment of Infectious Diseases, The First Affiliated Hospital, Zhejiang University School of Medicine, Hangzhou, China; 3 Frontage Laboratories Inc., Suzhou, Jiangsu, China

**Keywords:** *Lactobacillus plantarum* GUANKE, allergic rhinitis, IgE, Th2 cytokines, treatment

## Abstract

**Background:**

Current treatment strategies for Allergic rhinitis (AR) are often limited by adverse effects and less efficacy. This study aims to evaluate the therapeutic effect of *Lactobacillus plantarum* GUANKE in either ragweed pollen (RAGW)- or house dust mite (HDM)-induced AR models.

**Methods:**

Mice were sensitized intraperitoneally with RAGW or HDM on days 0, 7, and 14, followed by daily intranasal challenges with the respective allergen from day 21 to day 49. The treatment group mice were orally administered cetirizine (10 mg/kg) as positive control or three different doses of *L. plantarum* GUANKE (10^6^, 10^8^, or 10^1^⁰ CFU/mouse) after daily intranasal challenges. From day 21 to day 49, nose-wiping and sneezing frequencies in mice were recorded at 4-day intervals (days 21, 25, 29, 33, 37, 41, 45, 49). The area under the curves (AUC) of nose-wiping frequency, sneezing frequency and their combined frequency was calculated across these time points. On day 50, mice were euthanized for sample collection. Serum IgE levels and Th2 cytokines (IL-4, IL-5, and IL-13) levels in both serum and nasal mucosa were measured by ELISA. Nasal mucosa tissues were also collected for histopathological analysis.

**Results:**

In both RAGW- and HDM-induced AR models, *L. plantarum* GUANKE significantly reduced the nose-wiping frequency, sneezing frequency, and their combined frequency in AR mice on day 49 and markedly reduced the AUCs of these frequencies from day 21 to day 49. *L. plantarum* GUANKE also significantly decreased serum IgE levels and Th2 cytokines (IL-4, IL-5, and IL-13) levels in both serum and nasal mucosa. Moreover, histopathological analysis showed that *L. plantarum* GUANKE significantly reduced nasal mucosal thickness, eosinophil infiltration, and goblet cell proliferation.

**Conclusion:**

This study demonstrated the *L. plantarum* GUANKE can alleviate AR symptoms induced by RAGW or HDM in mice by inhibiting the levels of IgE and Th2 cytokines as well as restoring the histopathological changes of nasal mucosa.

## 1 Introduction

Allergic rhinitis (AR) is an inflammatory disease of the nasal mucosa caused by immunoglobulin E (IgE)-mediated reactions after exposure to allergens such as pollen, dust mites, or animal dander (1). It is one of the world’s most common chronic illnesses among children and young adults, which is characterized by a stuffy nose, itchy nose, sneezing, runny nose, etc. (2, 3). The etiology of AR is influenced by multiple factors such as genetics, epigenetics, and environment as well (4). AR is classified as type I allergic disease, and its typical feature is the increase of serum IgE level (5), which is caused by the overactivation of T helper 2 (Th2) cells immune response in susceptible individuals after exposure to the allergen. The Th2 cells synthesize high levels of Th2 cytokines such as interleukin (IL)-4, IL-5, and IL-13, which induces IgE overproduction by plasma cells, leading mast cells to degranulate and release inflammatory mediators such as histamine and cysteinyl-leukotrienes (6, 7). Clinically this results in the typical symptoms of rhinorrhea, sneezing, itching and nasal blockage. Currently, the treatments for AR patients mainly include antihistamines, nasal decongestants, glucocorticoids and expensive immunotherapy, which are characterized by a higher incidence of adverse effects (e.g., dry mouth, drowsiness and dizziness) and a limited therapeutic benefit (8–11). Therefore, there is an urgent need to develop safer and more effective therapeutic approaches for AR.

Probiotics are live microorganisms that, when administered in appropriate amounts, can have beneficial effects on the health of the host. In recent years, several studies have shown that the *Lactobacillus* species could treat and prevent AR by activating the immune system and regulating the immune response in respiratory allergic diseases (12–16). The GUANKE strain belongs to *Lactobacillus plantarum*, originally isolated from the fecal sample of a healthy individual (17). This study aimed to investigate the therapeutic potential of *Lactobacillus plantarum* GUANKE for AR in mice, which are induced by either ragweed pollen (RAGW) or house dust mites (HDM), two common allergens for seasonal and perennial AR, respectively (18, 19).

## 2 Materials and methods

### 2.1 Reagents

Reagents used in the study were obtained from the following sources: Ragweed pollen extract (RM56, Beijing Bored International Trading Co., Ltd), house dust mite extract (XPB82D3A25, GREER), and aluminum hydroxide (vac-alu-50, InvivoGen). Cetirizine (HY-17042, MCE) was used as a positive control for this study. ELISA kits used in the study included: mouse IgE uncoated ELISA kit (88-50460-88, Invitrogen), mouse HDM IgE ELISA kit (YPGX0374, Youpin Biotechnology). high sensitivity mouse IL-4 ELISA kit (E-HSEL-M0002, Elabscience), mini sample mouse IL-5 ELISA kit (E-MSEL-M0046, Elabscience), and mouse IL-13 ELISA kit (E-EL-M0727, Elabscience). Histochemical staining agents used in the study included: Hematoxylin (H810910, Shanghai Hansi Chemical Co., Ltd.), Eosin (LG-DH0045, Shanghai Yuduo Biotechnology Co., Ltd.), Chromogenic acid staining solution (S191072, Pinofei Biotechnology Co., Ltd), and PAS staining kit (S191008, Pinofei Biotechnology Co., Ltd).

### 2.2 Bacterial culture

The *Lactobacillus plantarum* GUANKE strain was cultured in Man-Rogosa-Sharpe broth at 37 °C in a CO_2_ Incubator. Bacteria in the logarithmic phase of growth were washed with phosphate-buffered saline (PBS), and the bacterial sludge was preserved directly at −20 °C for less than 5 days. Before use, the stored sludge was serially diluted with PBS, plated onto Man-Rogosa-Sharpe Agar, and incubated in a CO_2_ Incubator for 24 h. The colonies were then counted to calculate the bacterial concentration (CFU/g). Before oral administration to mice, the bacterial sludge was resuspended in sterile PBS and diluted to the desired concentration.

### 2.3 Animals

Six-week-old female BALB/c mice were purchased from Zhejiang Weitonglihua Laboratory Animal Technology Co, Ltd. (Zhejiang, China). They were housed in plastic cages at a controlled temperature of 23 ± 3 °C and a relative humidity of 60% ± 20%, with a 12-h dark/light cycle throughout the study. Food and aseptic tap water were provided *ad libitum*. All animals were acclimated for 7 days before the initiation of the experiments.

### 2.4 Ethics statement

All experimental procedures used in the study were approved by the Experimental Animal Ethics Committee of the Frontage Laboratories Inc. and conducted in accordance with the National Institutes of Health Guidelines for the Care and Use of Laboratory Animals (approval numbers: AN-2024-S186; AN-2024-S187; AN-2024-S188).

### 2.5 Experimental AR establishment and treatment

**Figure 1:**
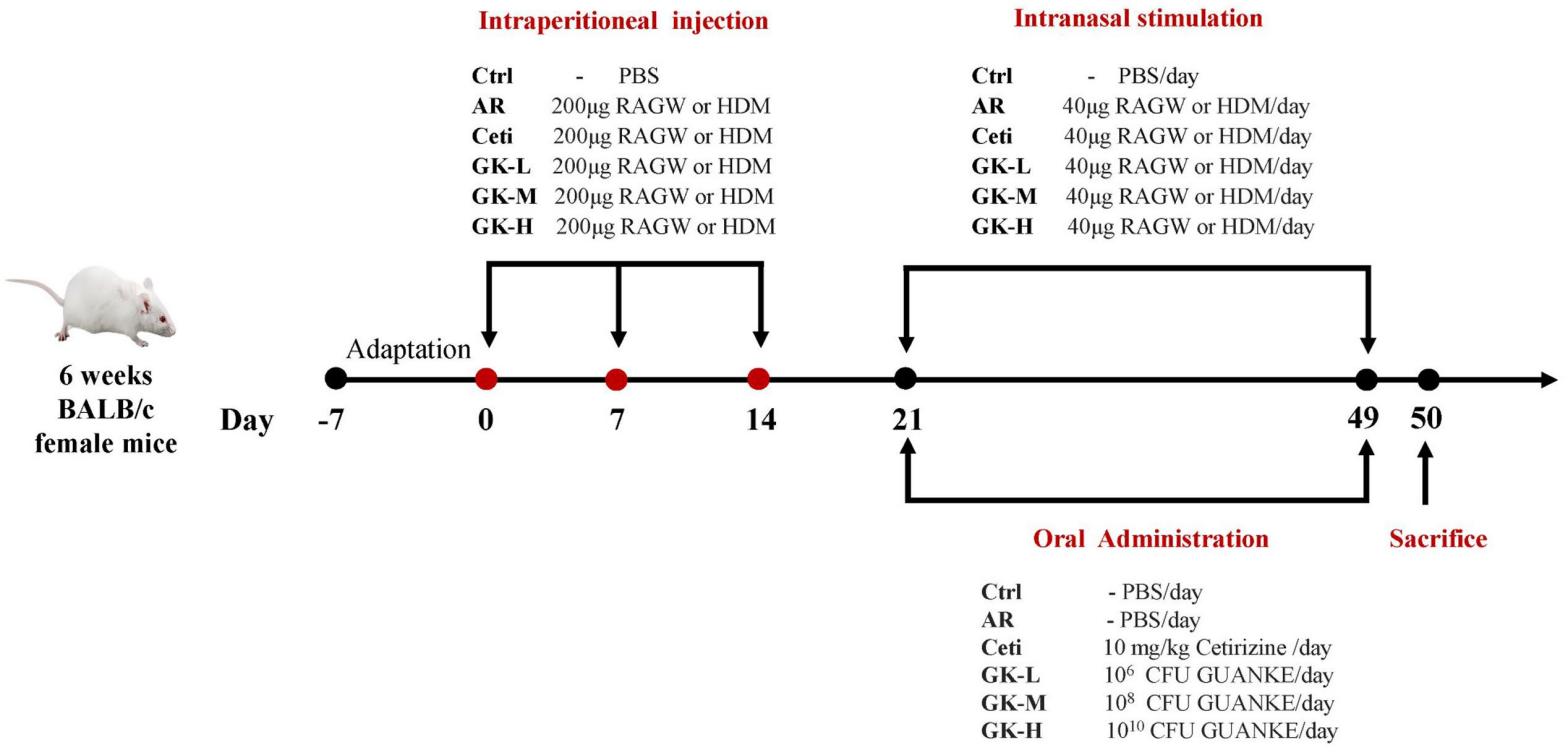
Illustration of the experimental procedure for AR mouse model induced by either RAGW or HDM. RAGW, ragweed pollen; HDM, house dust mite.

We established two AR mouse models induced by either RAGW or HDM. Each AR model involved 60 BALB/c mice, which were randomly divided into six groups (*n* = 10 per group) as follows: (1) normal control group (Ctrl), (2) AR model group (AR), (3) AR group treated with Cetirizine (Ceti), (4) AR group treated with 10^6^ CFU of GUANKE strain (GK-L), (5) AR group treated with 10^8^ CFU of GUANKE strain (GK-M), (6) AR group treated with 10^10^ CFU of GUANKE strain (GK-H). Figure 1 summarizes the sensitization methods of either RAGW or HDM, as well as the treatment schedule of the GUANKE strain. On days 0, 7, and 14, the Ctrl group mice were intraperitoneally injected with 200 μL PBS; other mouse groups were intraperitoneally injected with 200 μL PBS containing 200 μg of either RAGW or HDM and 2 mg of aluminum hydroxide. From day 21 to day 49, all mice in each experimental group were locally sensitized through daily intranasal challenges, with 10 μL PBS being dropped into each nostril in the Ctrl group and 10 μL PBS containing 20 μg either RAGW or HDM being dropped into each nostril in other mouse groups. After daily intranasal challenges, the AR groups treated were orally administered either 200 μL Ceti (10 mg/kg) or three different doses of GUANKE strain (10^6^, 10^8^, or 10^10^ CFU /mouse) once a day, while the Ctrl group and the untreated AR group were orally administered 200 μL of PBS. On day 50, serum and nasal mucosal samples were collected from each mouse under anesthesia for further analysis.

### 2.6 Assessments of nasal symptoms

Starting from the 21st day of the experiment, nasal symptoms of each mouse were recorded at 4-day intervals (i.e., on days 21, 25, 29, 33, 37, 41, 45 and 49). Specifically, the frequencies of nose-wiping and sneezing in each mouse group were observed and recorded within 15 min after intranasal challenge. A randomized and double-blind approach was rigorously employed to ensure the objectivity and reliability of the observational data.

### 2.7 Detection of IgE and Th2 cytokines in serum

Blood samples were collected from mouse hearts, and centrifuged at 3,000 rpm for 10 min at 4 °C to collect the serum. The expression levels of IgE, IL-4, IL-5, and IL-13 in serum were assessed by using appropriate ELISA kit according to the manufacturer’s instructions of each kit.

### 2.8 Detection of Th2 cytokines in nasal mucosa

The nasal mucosa was collected and homogenized in 500 μL of pre-cooled PBS containing 1% protease inhibitor on ice, and centrifuged at 13,000 × *g* for 10 min at 4 °C. The levels of Th2 cytokines (IL-4, IL-5, and IL-13) were assessed with appropriate ELISA kit per manufacturer’s instruction of each kit.

### 2.9 Histopathological analyses

Nasal tissues were collected for histopathological observation. The head of the mouse was removed and their lower jaw, skin, and soft tissue were discarded. The remaining nasal mucosa wrapped in the nasal and skull bone was fixed in 10% neutral formalin fixing for 24 h. After fixation, the nasal and skull bone was decalcified, paraffin-embedded, and sliced. Hematoxylin and eosin (H&E) staining were used to analyze nasal mucosal thickness. Periodic acid-Schiff (PAS) staining and Chromogenic acid staining were used to analyze eosinophil infiltration and goblet cell proliferation, respectively. The slides were scanned with slide digital scanners (Pannoramic SCAN II, Jinan Dangier Electronic Co., Ltd.) and read using Aperio ImageScope x64 software (v. 2.4.0.504, Leica Biosystems Imaging, Inc.). A randomized and double-blind approach was rigorously adopted in the histopathological evaluation of nasal mucosa.

### 2.10 Statistical analysis

Statistical analysis was performed using GraphPad Prism 8 (GraphPad, La Jolla, CA, USA). Data are expressed as Mean ± Standard Error (SEM). Repeated measures analysis of variance (ANOVA) was used to compare the frequency of AR symptoms among groups at different time points. For data of other indicators, statistical comparisons were made by using one-way ANOVA test, followed by a multiple comparison test with Dunnett’s test. The statistical significance was set at *p* < 0.05.

## 3 Results

### 3.1 GUANKE strain effectively relieves AR symptoms in mice induced by either RAGW or HDM

**Figure 2:**
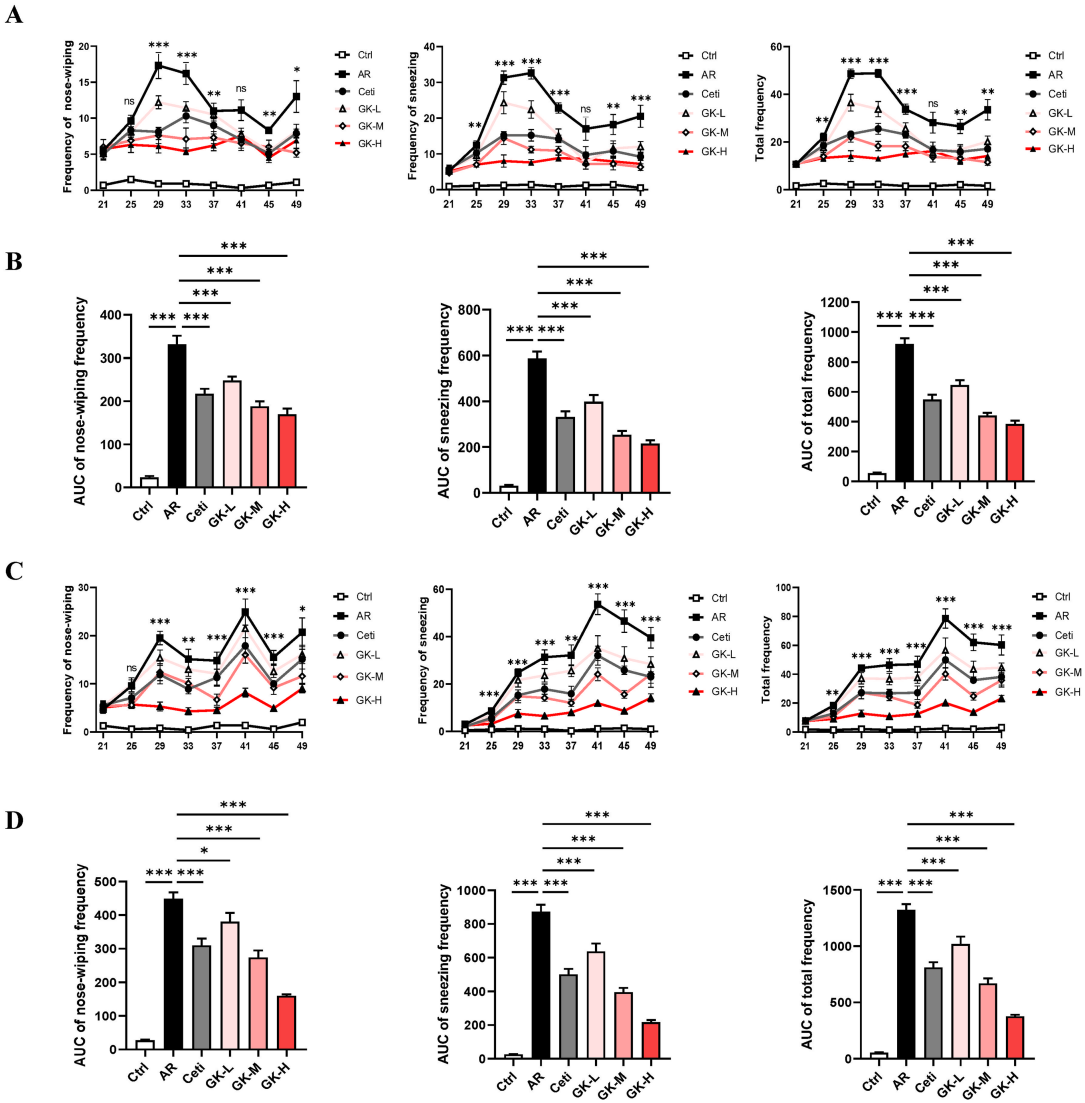
The effect of GUANKE strain on AR symptoms induced by either RAGW or HDM in mice. From day 21 to day 49, the nose-wiping frequency, sneezing frequency, and their combined frequency in the RAGW-induced AR model **(A)** or HDM-induced AR model **(C)** (*n* = 10). The “*” or “ns” in **A,C** represents the *p*-value of the AR group compared to the GK-H group. From day 21 to day 49, the AUC of nose-wiping frequency, sneezing frequency, and their combined frequency in the RAGW-induced AR model **(B)** or HDM-induced AR model **(D)** (*n* = 10). The data are presented as the mean ± SEMs. ns, not significant; *p < 0.05; **p < 0.01; ***p < 0.001. RAGW, ragweed pollen; HDM, house dust mite; Ctrl, control; AR, allergic rhinitis; Ceti, Cetirizine; GK-L, low-dose GUANKE (10^6^ CFU); GK-M, middle-dose GUANKE (10^8^ CFU); GK-H, high-dose GUANKE (10^10^ CFU).

Nose-wiping and sneezing behavior are the primary symptoms of AR (20). To assess the severity of AR symptoms, the frequencies of nose-wiping and sneezing within 15 min after intranasal challenges were recorded for each mouse. As shown in Figures 2A,C, in both RAGW- and HDM-induced AR models, from day 25 to day 49, the nose-wiping frequency, sneezing frequency, and their combined frequency in the AR group were significantly higher than in the control (Ctrl) group (*p* < 0.001), confirming the successful establishment of AR mouse models induced by either RAGW or HDM. On the other hand, compared with the AR group, in the RAGW-induced AR model, high-dose *L. plantarum* GUANKE significantly reduced the sneezing frequency, nose-wiping frequency, and their combined frequency in a dose-dependent manner on days 29, 33, 37, 45, and 49 (*p* < 0.05). Notably, on day 41, the reductions of nose-wiping and sneezing frequencies in mice did not reach statistical significance (*p* > 0.05), which might be related to the tolerance of mice to RAGW allergen and GUANKE strain. In contrast, in the HDM-induced AR model, treatment with high dose *L. plantarum* GUANKE significantly reduced the nose-wiping frequency, sneezing frequency, and their combined frequency in a dose-dependent manner from day 29 to day 49 (*p* < 0.01), with the high-dose *L. plantarum* GUANKE being the more effective than cetirizine in relieving AR symptoms. Interestingly, in both RAGW- and HDM-induced AR models, on day 25, high-dose *L. plantarum* GUANKE significantly reduced the sneezing frequency compared with the AR group (*p* < 0.01), but there was no statistically significant decrease in the frequency of nose-wiping of mice (*p* > 0.05). This suggests that the relief of nose-wiping symptom in mice might require a longer treatment time.

To comprehensively evaluate the therapeutic effects of each treatment, the area under the curves (AUC) for nose-wiping frequency, sneezing frequency, and their combined frequency over the 28-day treatment period was analyzed. As shown in Figures 2B,D, compared with the Ctrl group, the AR group showed a significant increase in all three AUC metrics from day 21 to day 49 (*p* < 0.001). In contrast, mice treated with cetirizine (Ceti), low-dose GUANKE (GK-L), medium-dose GUANKE (GK-M), or high-dose GUANKE (GK-H) exhibited significantly lower AUC values for all symptoms compared to the AR group (*p* < 0.05), and the most significant effect occurred in the GK-H group (*p* < 0.001). These results collectively demonstrate that *L. plantarum* GUANKE significantly alleviates AR symptoms—namely nose-wiping and sneezing—in AR mice sensitized with either RAGW or HDM, with the GK-H group exhibiting the most pronounced therapeutic effect.

### 3.2 GUANKE strain reduces IgE in the serum of AR mice induced by either RAGW or HDM

**Figure 3:**
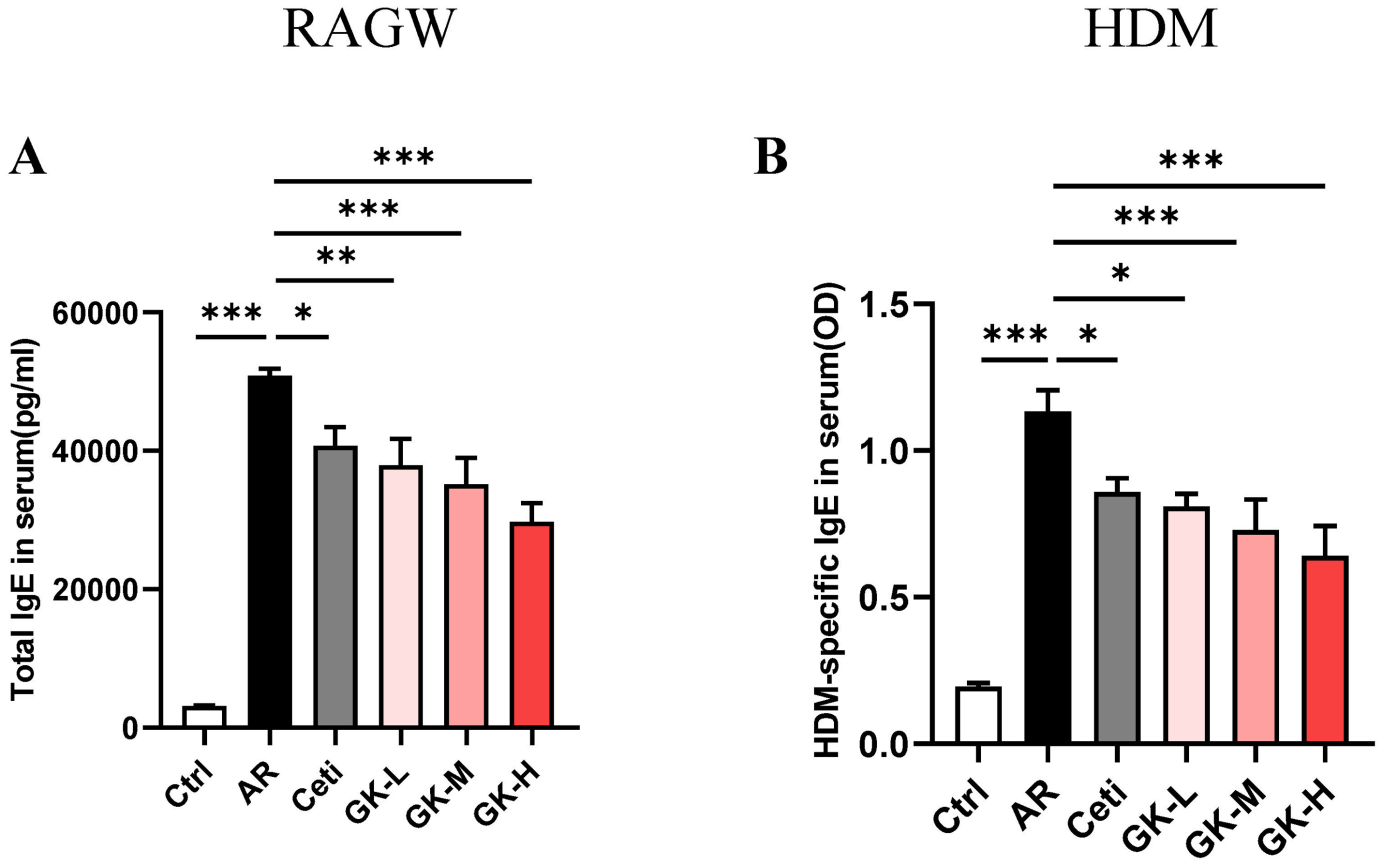
The effect of GUANKE strain on IgE in the serum of AR mice induced by either RAGW or HDM. **(A)** The total IgE levels in serum of each group of mice in RAGW-induced AR model (*n* = 10). **(B)** The HDM-specific IgE levels in serum of each group of mice in HDM-induced AR model (*n* = 10). The data are presented as the mean ± SEMs. *p < 0.05; **p < 0.01; ***p < 0.001. RAGW, ragweed pollen; HDM, house dust mite; Ctrl, control; AR, allergic rhinitis; Ceti, Cetirizine; GK-L, low-dose GUANKE (10^6^ CFU); GK-M, middle-dose GUANKE (10^8^ CFU); GK-H, high-dose GUANKE (10^10^ CFU).

AR is characterized by the increased production of serum IgE (21). In the RAGW-induced AR model, the total IgE level in the serum of the AR group was significantly increased compared with the Ctrl group (*p* < 0.001). However, the total IgE level in the serum of the Ceti group, GK-L group, GK-M group, and GK-H group were significantly reduced (*p* < 0.05) with the most significant effect occurred in the GK-H group (*p* < 0.001) (Figure 3A). Similarly, in the HDM-induced AR model, the HDM-specific IgE level in serum of the AR group was significantly increased compared with the Ctrl group (*p* < 0.001). The HDM-specific IgE levels in the serum of the Ceti group, GK-L group, GK-M group, and GK-H group were significantly reduced compared with the AR group (*p* < 0.05) with the most significant effect occurring in the GK-H group (*p* < 0.001) (Figure 3B). These results indicate that treatment with GUANKE strain reduced the level of IgE in serum of AR mice induced by either RAGW or HDM, and the GK-H group showed the most significant therapeutic effect.

### 3.3 GUANKE strain inhibits the production of Th2 cytokines in the serum of AR mice induced by either RAGW or HDM

**Figure 4:**
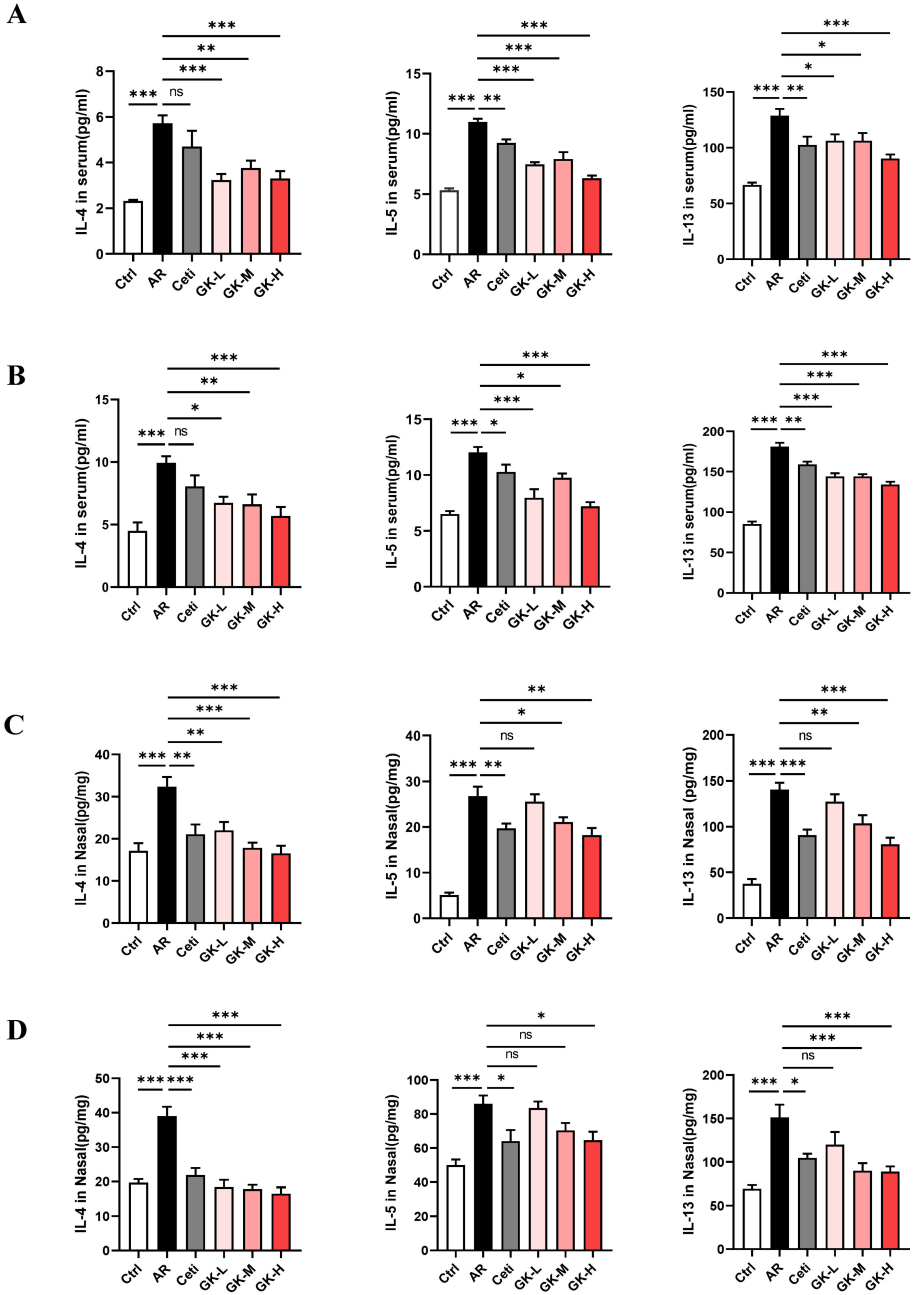
The effect of GUANKE strain on the levels of Th2 cytokines in the serum and nasal mucosa of AR mice induced by either RAGW or HDM. The levels of Th2 cytokines in the serum in the RAGW-induced AR model **(A)** and HDM-induced AR model **(B)** (*n* = 10). The levels of Th2 cytokines in the nasal mucosa in the RAGW-induced AR model **(C)** and HDM-induced AR model **(D)** (*n* = 5). The data are presented as the means ± SEMs. ns, not significant; *p < 0.05; **p < 0.01; ***p < 0.001. RAGW, ragweed pollen; HDM, house dust mite; Ctrl, control; AR, allergic rhinitis; Ceti, Cetirizine; GK-L, low-dose GUANKE (10^6^ CFU); GK-M, middle-dose GUANKE (10^8^ CFU); GK-H, high-dose GUANKE (10^10^ CFU).

IL-4, IL-5, and IL-13 are key Th2 cytokines that play pivotal roles in IgE production and eosinophil activation in AR. As shown in Figures 4A,B, in both RAGW- and HDM-induced AR models, the levels of IL-4, IL-5, and IL-13 were significantly elevated in the AR group compared with the Ctrl group (*p* < 0.001), indicating a robust Th2-mediated inflammatory response.

Compared with the AR group, the Ceti group exhibited a significant reduction in IL-5 and IL-13 levels (*p* < 0.05); however, the reduction in IL-4 was not statistically significant (*p* > 0.05). In contrast, treatment with *L. plantarum* GUANKE at all tested doses significantly decreased the levels of IL-4, IL-5, and IL-13 (*p* < 0.05), with the most pronounced effect observed in the GK-H group (*p* < 0.001). These results suggest that GUANKE treatment effectively suppresses Th2 cytokines production and that the high-dose GUANKE regimen exerts the strongest therapeutic effect in modulating the Th2 immune response in AR.

### 3.4 GUANKE strain inhibits the production of Th2 cytokines in the nasal mucosa of AR mice induced by either RAGW or HDM

The levels of Th2 cytokines in the nasal mucosa were also assessed. As shown in Figures 4C,D, in both RAGW- and HDM-induced AR models, the levels of IL-4, IL-5, and IL-13 in the AR group were significantly elevated compared with those in the Ctrl group (*p* < 0.001), indicating an enhanced local Th2-type inflammation.

Compared with the AR group, treatment with Cetirizine (Ceti) and high doses of *L. plantarum* GUANKE (GK-H) significantly reduced the levels of IL-4, IL-5, and IL-13 in the nasal mucosa (*p* < 0.05), with a more pronounced effect observed in the GK-H group. In the GK-L group, IL-4 levels were also significantly decreased (*p* < 0.01), while reductions in IL-5 and IL-13 levels did not reach statistical significance (*p* > 0.05). Interestingly, in the HDM-induced AR model, medium-dose GUANKE (GK-M) treatment significantly decreased IL-4 and IL-13 levels (*p* < 0.01), but the decrease in IL-5 levels did not reach statistical significance (*p* > 0.05). These findings suggest that *L. plantarum* GUANKE can effectively suppress Th2 cytokines expression in the nasal mucosa, particularly at higher doses, and that the GK-H group demonstrates the strongest anti-inflammatory effect.

### 3.5 GUANKE strain restores histopathological changes in nasal mucosal of AR mice induced by either RAGW or HDM

**Figure 5:**
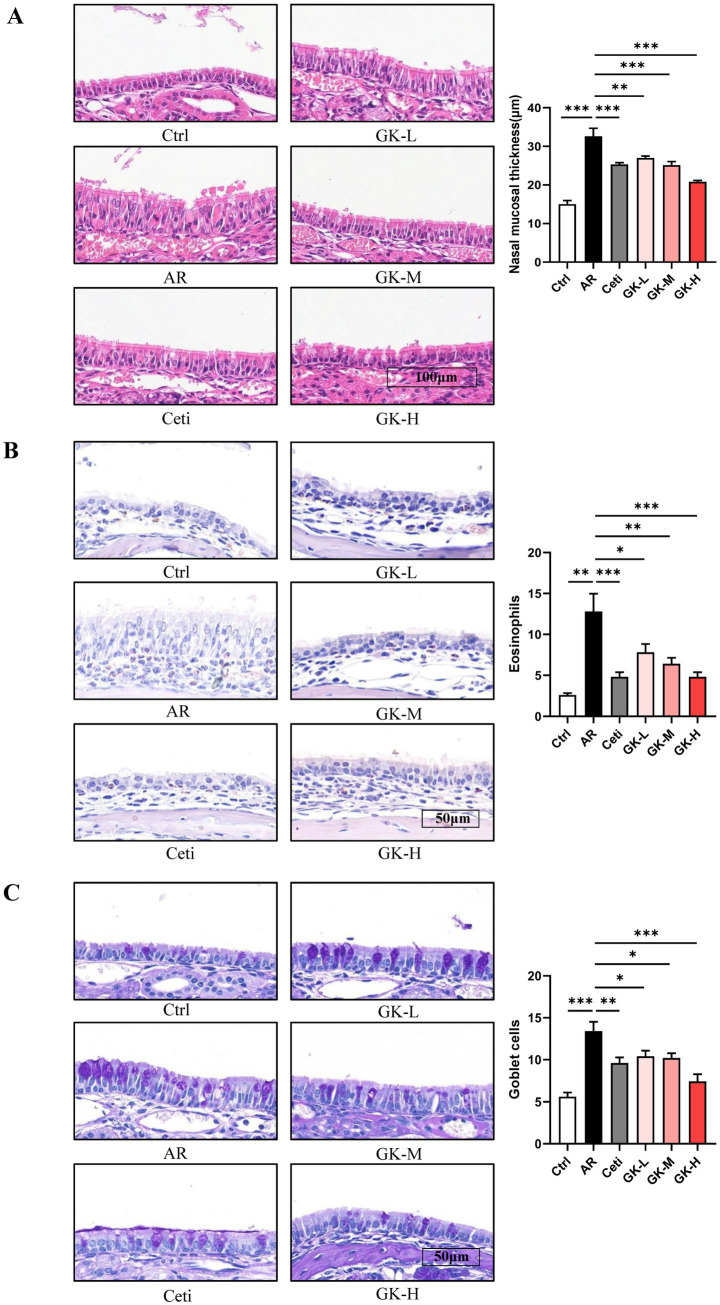
The effect of GUANKE strain on histopathological changes in nasal mucosa of AR mice induced by RAGW. **(A)** Representative H&E staining image of nasal mucosa (left) and quantification of nasal mucosal thickness (right). Magnification = 20 ×, scale bar = 100 μm; *n* = 5. **(B)** Representative chromogenic acid staining image (left) and eosinophil counts (right) of nasal mucosa. Magnification = 40 ×, scale bar = 50 μm; *n* = 5. **(C)** Representative PAS staining image (left) and goblet cell counts (right) of nasal mucosa. Magnification = 40 ×, scale bar = 50 μm; *n* = 5. The data are presented as the mean ± SEMs. *p < 0.05; **p < 0.01; ***p < 0.001. RAGW, ragweed pollen; Ctrl, control; AR, allergic rhinitis; Ceti, Cetirizine; GK-L, low-dose GUANKE (10^6^ CFU); GK-M, middle-dose GUANKE (10^8^ CFU); GK-H, high-dose GUANKE (10^10^ CFU).

**Figure 6:**
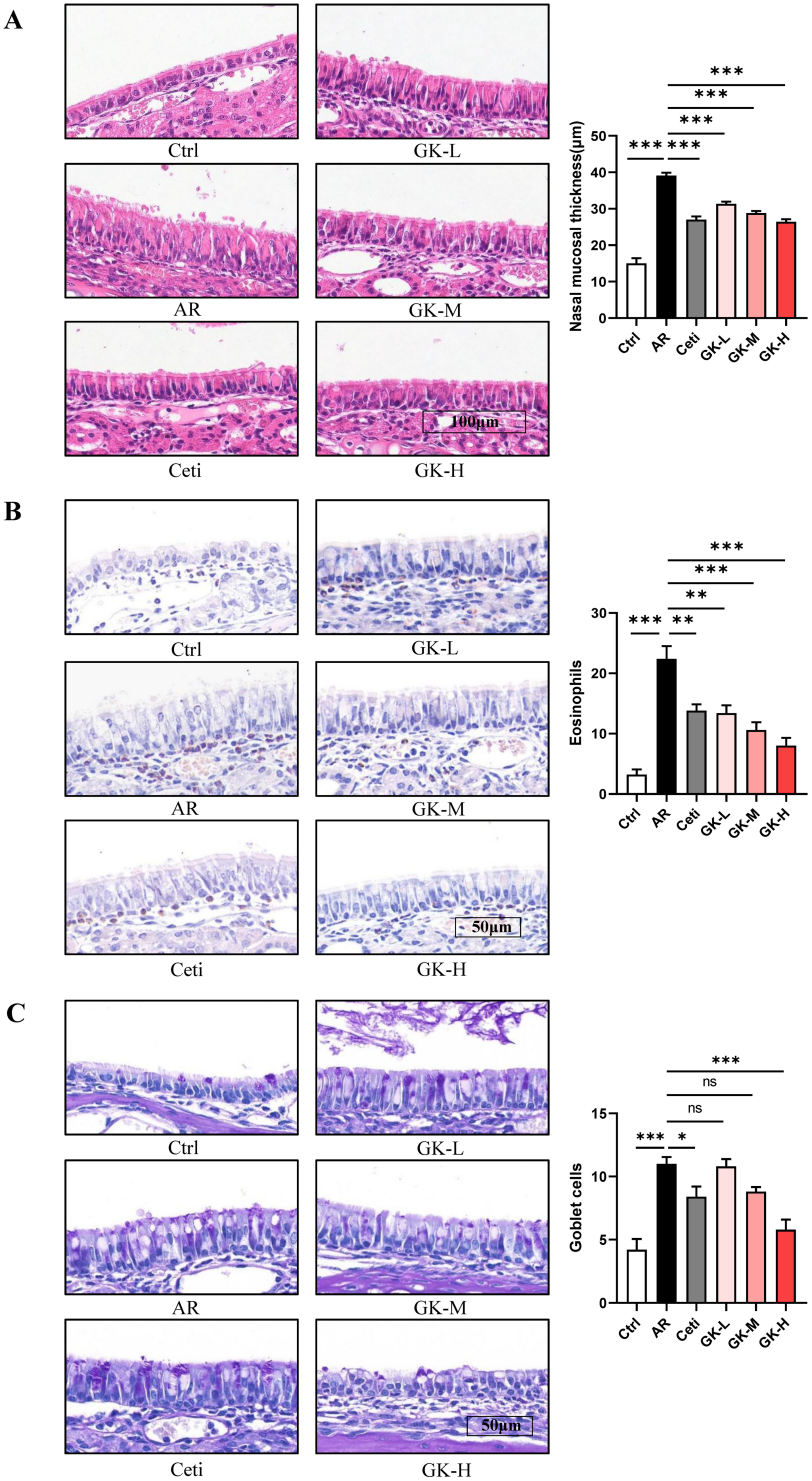
The effect of GUANKE strain on histopathological changes in nasal mucosa of AR mice induced by HDM. **(A)** Representative H&E staining image of nasal mucosa (left) and quantification of nasal mucosal thickness (right). Magnification = 20 ×, scale bar = 100 μm; *n* = 5. **(B)** Representative chromogenic acid staining image (left) and eosinophil counts (right) of nasal mucosa. Magnification = 20 ×, scale bar = 100 μm; *n* = 5. **(C)** Representative PAS staining image (left) and goblet cell counts (right) of nasal mucosa. Magnification = 20 ×, scale bar = 100 μm; *n* = 5. The data are presented as the mean ± SEMs. ns, not significant; *p < 0.05; **p < 0.01; ***p < 0.001. HDM, house dust mite; Ctrl, control; AR, allergic rhinitis; Ceti, Cetirizine; GK-L, low-dose GUANKE (10^6^ CFU); GK-M, middle-dose GUANKE (10^8^ CFU); GK-H, high-dose GUANKE (10^10^ CFU).

Histopathological changes in nasal mucosa can reflect the severity of AR (22). To evaluate these changes, H&E staining, chromogenic acid staining, and PAS staining were used to assess nasal mucosal thickness, eosinophil infiltration, and goblet cell proliferation, respectively. In both RAGW- and HDM-induced AR models, H&E staining revealed a significant increase in nasal mucosal thickness in the AR group compared with the Ctrl group (*p* < 0.001). Treatment with Cetirizine (Ceti), low (GK-L), medium (GK-M), and high (GK-H) doses of *L. plantarum* GUANKE significantly reduced nasal mucosal thickness compared with the AR group (*p* < 0.01), with the GK-H group showing the most pronounced improvement (*p* < 0.001) (Figures 5A, 6A). Chromogenic acid staining showed a significant increase in eosinophil infiltration in the AR group compared with the Ctrl group (*p* < 0.01). Treatment with Ceti, GK-L, GK-M, and GK-H significantly reduced eosinophil infiltration (*p* < 0.05), again with the GK-H group demonstrating the most significant reduction (*p* < 0.001). (Figures 5B, 6B). PAS staining revealed a marked increase in goblet cell proliferation in the AR group compared with the Ctrl group (*p* < 0.001). This increase was significantly attenuated in the Ceti, GK-L, GK-M, and GK-H groups in the RAGW- induced AR models (*p* < 0.05), with the GK-H group showing the greatest effect (*p* < 0.001). In the HDM-induced AR model, the proliferation of goblet cell was significantly reduced in the Ceti group and GK-H group (*p* < 0.05), but no statistically significant decrease in goblet cell proliferation was observed in the GK-L and GK-M groups compared to the AR group (*p* > 0.05) (Figures 5C, 6C). Taken together, these results demonstrate that treatment with *L. plantarum* GUANKE effectively ameliorates key histopathological features of AR, including mucosal thickening, eosinophil infiltration, and goblet cell proliferation, with the high-dose (GK-H) treatment yielding the most robust therapeutic outcomes.

## 4 Discussion

The typical clinical symptoms of AR are sneezing, nasal mucus, nasal itching, etc. (23). In this study, the therapeutic effect of *L. plantarum* GUANKE was evaluated based on the nose-wiping and sneezing behaviors of mice. The results showed that in both RAGW- and HDM-induced AR models, *L. plantarum* GUANKE significantly reduced the nose-wiping frequency, sneezing frequency, and their combined frequency in AR mice on day 49. And it also markedly reduced the AUCs of these frequencies in a dose-dependent manner from day 21 to day 49, with the high-dose GUANKE being the more effective than cetirizine in relieving AR symptoms (Figure 2). These results indicate that *L. plantarum* GUANKE can effectively improve the AR symptoms induced by either RAGW or HDM in mice.

The imbalance between Th1- and Th2-mediated immune responses is considered the primary cause of AR (24). When initially exposed to allergens, sensitized individuals exhibit an overactive Th2 immune response. Activated Th2 cells secrete cytokines like IL-4 and IL-13, promoting the transformation of B cells into plasma cells to produce IgE that subsequently binds to high-affinity IgE receptors on mast cells. Upon re-exposed to the same allergen, the binding of the allergen to IgE on mast cells can cause degranulation of mast cells and release of inflammatory mediators, leading to AR symptoms (6). Therefore, the typical feature of AR is an increase in serum IgE level, commonly regarded as a biomarker for AR (25). Furthermore, IL-4 promotes mast cell differentiation and maturation, while IL-5 plays a central role in eosinophil activation, maturation, and prolonged survival (26). In our study, treatment with *L. plantarum* GUANKE significantly reduced the serum IgE level and the levels of Th2 cytokines (IL-4, IL-5, and IL-13) in both the serum and nasal mucosa of AR mice induced by RAGW or HDM (Figures 3, 4). These findings suggest that *L. plantarum* GUANKE promotes the balance of Th1 and Th2 by inhibiting the overactivation of Th2 cells, thereby reducing IgE production.

Concurrently, during AR progression, under the stimulation of Th2 cytokines and inflammatory mediators, eosinophils are activated and infiltrate into the nasal mucosa, leading to nasal mucosal swelling and inflammation (27). This stimulation can promote the proliferation of goblet cells, leading to excessive mucus secretion, which plays a crucial role in the severity of AR (28). The present study revealed that *L. plantarum* GUANKE markedly improved the histopathological changes of the nasal mucosa, including nasal mucosal thickening, eosinophil infiltration, and goblet cell proliferation, which contributes to the alleviation of AR symptoms (Figures 5, 6).

These findings collectively suggest that *L. plantarum* GUANKE reduces IgE production and alleviates nasal mucosal inflammation in mice by suppressing the generation of Th2 cytokines (IL-4, IL-5, and IL-13), ultimately alleviate AR symptoms in mice. Similar to the results of this study, Zhou et al. (29) demonstrated that oral administration of *Lacticaseibacillus paracasei* GOLDGUT-Lpc969 can also alleviate AR symptoms in mice by rebalancing the Th1/Th2 ratio through decreasing Th2 cytokines production, thus inhibiting the production of IgE. However, the mechanism by which *L. plantarum* GUANKE inhibits the Th2 inflammatory response remain to be further investigated. It has been reported that type 1 helper T (Th1) cells play a critical role in alleviating AR symptoms by secreting anti-inflammatory cytokines such as IFN-γ, TNF-α and IL-2, thereby suppressing Th2 immune response (30). For instance, Choi et al. (31) reported that *Lactobacillus plantarum* CJLP133 and CJLP243 enhanced Th1 immune response in mice, leading to increased IFN-γ secretion and inhibition of Th2 cytokines (IL-4, IL-5, and IL-13), thus restoring Th1/Th2 balance. Besides, regulatory T cells (Tregs) are also crucial in inhibiting the activity of Th2 cells. Tregs are the mediators of immunological tolerance and possess anti-inflammatory capabilities (32). They can inhibit the differentiation of Th0 (a common precursor of Th1 and Th2 cells) into Th2 cells (33, 34). Ren et al. (35) found that oral administration of *Bifidobacterium breve* can regulate Th1/Th2 balance by inducing CD4+CD25+Tregs activity and inhibiting Th2 immune response without evoking the Th1 response. Moreover, there is a close interaction between gut microbiota and the host immune system (36). AR is associated with imbalanced gut microbiota (37). The gut microbiota disturbance stimulates the immune cells such as macrophages, dendritic cells, eosinophils, and T cells, resulting in inflammation including colitis and rhinitis (38). Kim et al. (39) found that *Bifidobacterium longum* IM55 and *Lactobacillus plantarum* IM76 can alleviate AR symptoms in mice by restoring gut microbiota disturbance and Th2/Treg imbalance. Probiotics can also exert anti-allergic effects through producing specific metabolites to inhibit the Th2 immune response. Zhang et al. (40) found that *Limosilactobacillus reute*ri significantly increased the production of flavonoid compounds in the intestine, notably luteolin, which can alleviate AR symptoms in mice by balancing Th1/Th2 cells. Based on existing research on probiotic treatment of AR mice, *L. plantarum* GUANKE is likely to alleviate AR symptoms by inhibiting Th2 immune response through the above mechanisms, which requires further in-depth research to explore.

In recent years, there have been multiple clinical translational studies on probiotic intervention in AR. The NVP-1703, a mixture of *Bifidobacterium longum* and *Lactobacillus plantarum*, significantly improved total nasal symptom score (TNSS), nasal symptom duration score (NSDS) and quality of life (QoL) in children with perennial AR, accompanied by decreases in the ratios of Th2 cytokines to IL-22 (41). Another clinical study showed that the combination of probiotics (*Bifidobacterium longum* G301, *Bifidobacterium infantis* G201, *Lactobacillus acidophilus* G80, *Lactobacillus paracasei* G110, *Lacto-bifidobacterium* G101 and *Lactobacillus gasseri* G12) and prebiotics (fructo-oligosaccharide, galacto-oligosaccharide, inulin and xylo-oligosaccharide) administered for 90 days significantly reduced TNSS and the scores of rhinorrhea and sneezing of seasonal AR patients, which is related to increased levels of IFN-γ and fluctuations in the composition and metabolic function of the intestinal microbiota (42). Additionally, a systematic review and meta-analysis on the efficacy and safety of probiotics for AR showed that probiotics supplementation for patients with AR can ameliorate AR symptoms and improve the quality of life, especially for the strains of *Lactobacillus* or *Bifidobacterium*. Probiotic therapy for AR generally exhibits great safety, with only a small number of individuals experiencing symptoms such as diarrhea, abdominal pain, and/or bloating. However, these symptoms are usually mild and transient, and they can be resolved spontaneously without the need for medication (43). Although, in this study, we demonstrated the efficacy of *L. plantarum* GUANKE in treating AR in animal models, it is still necessary to conduct clinical studies to further understand the efficacy of *L. plantarum* GUANKE in treating AR induced by RAGW or HDM.

This study has certain limitations. First, the experimental animals were limited to mouse models, and their applicability in humans needs to be validated clinically. Second, it is suggested that *L. plantarum* GUANKE exerts anti-allergic effects by inhibiting Th2 immune responses, but the specific molecular mechanism has not been fully elucidated and further research is needed. Finally, although we showed that GUANKE can effectively treat AR symptoms induced by RAGW or HDM, it remains to be determined if GUANKE is effective as well for other allergens given so many allergens could cause AR in humans.

## 5 Conclusion

In this study, we demonstrated that in AR models induced by either RAGW or HDM in BALB/c mice, *L. plantarum* GUANKE significantly inhibited the production of IgE and Th2 cytokines (IL-4, IL-5, and IL-13), while also reducing eosinophil infiltration and goblet cell proliferation in the nasal mucosa. These effects contributed to the alleviation of AR symptoms of mice, suggesting GUANKE strain has the potential to become a therapeutic agent for AR.

## Data Availability

The raw data supporting the conclusions of this article will be made available by the authors, without undue reservation.
